# ZMYND12 serves as an IDAd subunit that is essential for sperm motility in mice

**DOI:** 10.1007/s00018-024-05344-7

**Published:** 2024-07-27

**Authors:** Chang Wang, Qingsong Xie, Xun Xia, Chuanying Zhang, Shan Jiang, Sihan Wang, Xi Zhang, Rong Hua, Jiangyang Xue, Haoyu Zheng

**Affiliations:** 1 College of Nursing, Anhui University of Chinese Medicine, Hefei, Anhui 230012 China; 2 Department of Gynaecology, The Affiliated Huai’an No. 1 People’s Hospital of Nanjing Medical University, Huai’an, Jiangsu 223300 China; 3 Department of Obstetrics and Gynecology, The First Affiliated Hospital of Anhui Medical University, Hefei, Anhui 230022 China; 4 The Central Laboratory of Birth Defects Prevention and Control, Ningbo Key Laboratory for the Prevention and Treatment of Embryogenic Diseases, Women and Children’s Hospital of Ningbo University, Ningbo, Zhejiang 315000 China; 5 Department of Reproductive Health and Infertility Clinic, The Affiliated Huai’an No. 1 People’s Hospital of Nanjing Medical University, Huai’an, Jiangsu 223300 China; 6 NHC Key Laboratory of Study on Abnormal Gametes and Reproductive Tract (Anhui Medical University), Hefei, Anhui 230032 China; 7 Key Laboratory of Population Health Across Life Cycle (Anhui Medical University), Ministry of Education of the People’s Republic of China, Hefei, Anhui 230032 China

**Keywords:** Spermatogenesis, Knockout mice, Male fertility, IDA, PRKACA

## Abstract

Inner dynein arms (IDAs) are formed from a protein complex that is essential for appropriate flagellar bending and beating. IDA defects have previously been linked to the incidence of asthenozoospermia (AZS) and male infertility. The testes-enriched ZMYND12 protein is homologous with an IDA component identified in *Chlamydomonas*. ZMYND12 deficiency has previously been tied to infertility in males, yet the underlying mechanism remains uncertain. Here, a CRISPR/Cas9 approach was employed to generate *Zmynd12* knockout (*Zmynd12*^*−/−*^) mice. These *Zmynd12*^*−/−*^ mice exhibited significant male subfertility, reduced sperm motile velocity, and impaired capacitation. Through a combination of co-immunoprecipitation and mass spectrometry, ZMYND12 was found to interact with TTC29 and PRKACA. Decreases in the levels of PRKACA were evident in the sperm of these *Zmynd12*^*−/−*^ mice, suggesting that this change may account for the observed drop in male fertility. Moreover, in a cohort of patients with AZS, one patient carrying a *ZMYND12* variant was identified, expanding the known AZS-related variant spectrum. Together, these findings demonstrate that ZMYND12 is essential for flagellar beating, capacitation, and male fertility.

**Supplementary Information:**

The online version contains supplementary material available at 10.1007/s00018-024-05344-7.

## Introduction

The motility of sperm is vital for male fertility owing to the need for sperm to propel themselves along the length of the female reproductive tract following ejaculation so that they can fertilize the egg [1]. Impaired sperm motility can directly result in male infertility [2]. Asthenozoospermia (AZS) is a common type of primary male infertility wherein patients exhibit < 40% motile spermatozoa or < 32% progressive spermatozoa despite the absence of any abnormalities in sperm morphology or sperm counts [3]. The genetic processes that govern sperm motility, however, remain incompletely understood.

As highly specialized cells, sperm consist of a head domain and a unique flagellum required for the oscillatory movement of these cells through the female reproductive tract such that they can fertilize mature oocytes [4]. The flagellar structure is highly conserved and consists of the cytoskeletal axoneme and a range of specifically organized peri-axonemal elements [5]. The axoneme is localized within the center of the flagella and is composed of nine peripheral doublet microtubules (DMTs) arranged around a central pair (CP) of microtubules in what has been termed a “9 + 2” arrangement [6]. Microtubule dynamics are shaped and maintained through the links that are formed among dynein arms, radial spokes, and the nexin-dynein regulatory complex (N-DRC) [7]. Variations in the many proteins present within sperm flagellum are thought to be closely associated with the pathogenesis of AZS in humans [8, 9]. The precise function of these proteins and how they contribute to mammalian infertility, however, has yet to be firmly established.

The swinging movement of sperm flagella is strongly dependent on dynein arm function, with axonemal dynein identification having yielded insight into the mechanistic basis for flagellar bending [10]. Axonemal dyneins are complex molecular motors composed of heavy (DHC), intermediate (IC), light (LC), and light intermediate chain (LIC) polypeptides with various molecular weights and activities [11]. Dynein motors are located within the axoneme, and are classified into the outer and inner dynein arms (ODAs and IDAs, respectively) [12]. Any form of axonemal dynein arm defects in *Chlamydomonas* has been demonstrated to result in the severe impairment of ciliary motility [13, 14]. In both mice and humans, biallelic male sterility-related mutations in several genes related to dynein arm component biosynthesis including *DNAH1* [15], *DNAH2* [16], *DNAH10* [17], and *DNALI1* [1], have been identified. While affected patients exhibit reductions in sperm movement and multiple morphological abnormalities of the flagella (MMAF), the specific genetic basis for AZS is incompletely understood.

ZMYND12 (Zinc Finger, MYND domain containing 12) is encoded on chromosomes 1 and 4 in humans and mice, respectively. The p38 homolog of ZMYND12 was first identified as an IDA component in *Chlamydomonas* [18]. Seven IDA subspecies (a-g) have been identified to date, each of which consists of one or two of eight distinct DHCs [19]. Prior studies identified p38 in *Chlamydomonas* as an IDAd-specific accessory subunit [20]. Coutton et al. recently found that 3 of 167 patients with MMAF harbored variants in the *ZMYND12* gene [21]. In line with its possible functional role in this context, knockdown of the ZMYND12 ortholog TbTAX-1 in *Trypanosoma brucei* had a pronounced effect on sperm motility [21, 22]. These results suggest a potential role for ZMYND12 deficiency in human AZS.

Advances in gene editing-based models have enabled the in vivo investigation of the distinct phenotypic effects associated with knockout and knockdown models [23]. Accordingly, a CRISPR/Cas9-based approach was herein used to generate *Zmynd12*-knockout mice as a means of exploring the phenotypic role played by ZMYND12 in vivo.

## Results

### Deletion of the testis-enriched ZMYND12 results in male subfertility

**Fig. 1:**
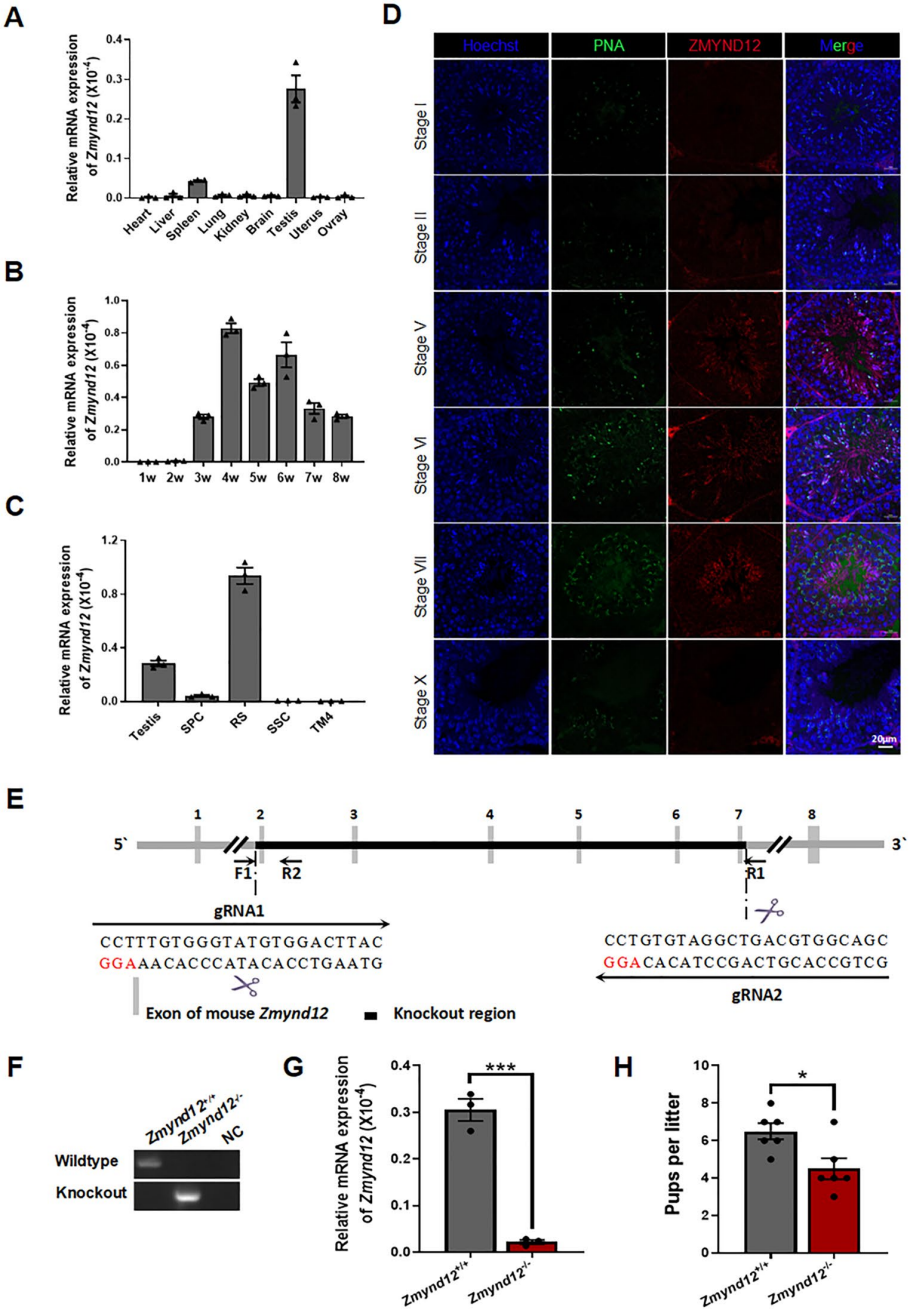
Sperm flagellin ZMYND12 deletion results in subfertility in male mice. (**A**) qPCR was used to detect the expression of *Zmynd12* in murine samples prepared from different tissues, with 18 S as a normalization control. Data are means ± SEM, *n* = 3. (**B**) *Zmynd12* mRNA levels in the testes of mice at different ages, with 18 S as a normalization control. W, weeks. Data are means ± SEM, *n* = 3. (**C**) qPCR was used to measure *Zmynd12* expression in male germ cells isolated from the testes of mice, with 18 S as a normalization control. Data are means ± SEM, *n* = 3. (**D**) IF staining for ZMYND12 (red) and PNA (green) in the testes of WT mice, *n* = 3. (**E**) Schematic overview of the approach to generating *Zmynd12*^−/−^ mice using a CRISPR/Cas9 approach. (**F**) PCR was used to identify murine genotypes with the F1, R1, and R2 primers. Wildtype and knockout mice were respectively identified using the F1/R1 and F1/R2 primer pairs. (**G**) qPCR was used to measure *Zmynd12* expression in the testes of *Zmynd12*^+/+^ and *Zmynd12*^−/−^ mice, with 18 S as a normalization control. Data are means ± SEM, *n* = 3. (**H**) Average numbers of pups per litter for male *Zmynd12*^+/+^ and *Zmynd12*^−/−^ mice. Data are means ± SEM, *n* = 3

Initially, murine ZMYND12 expression patterns across tissue types were analyzed via qPCR, revealing that it is expressed a high levels in the spleen and testis, with these levels being highest in the testis (Fig. 1A). Testis ZMYND12 mRNA levels initially began rising at 3 weeks postpartum and continued to rise with testis development into adulthood (Fig. 1B). When a STA-PUT (Sedimentation at Unit Gravity) approach was used for spermatocyte and spermatid isolation, the highest levels of *Zmynd12* enrichment were detected in the round spermatids (RS) (Fig. 1C). Subsequent immunofluorescent staining confirmed that ZMYND12 was present in the flagella of elongated spermatids in the testes, in addition to being present within spermatozoa from both humans and mice (Fig. 1D; Supplementary Fig. S1). These results suggest that ZMYND12 may play an important functional role in the flagellum.

As a protein with a high degree of evolutionary conservation, human and mouse ZMYND12 exhibit high similarity (Supplementary Fig. S2). In an effort to understand its functional role in vivo, a CRISPR/Cas9 strategy was used to generate *Zmynd12*-knockout (*Zmynd12*^*–/–*^) mice. For this approach, the zygotes of wild-type (*Zmynd12*^*+/+*^) mice were microinjected with Cas9 and gRNAs targeting exons 2–7 of *Zmynd12* (Fig. 1E). Subsequent PCR analyses confirmed the deletion of a 17,162 bp segment of the *Zmynd12* gene in the resultant mice (Fig. 1F). Quantitative PCR additionally confirmed that the *Zmynd12* was absent from the testes of *Zmynd12*^*–/–*^ mice, indicating that it had been successfully deleted (Fig. 1G).

The *Zmynd12*^*–/–*^ mice exhibited healthy growth with no evidence of any defects (Supplementary Fig. S3). When they were subjected to fertility testing, vaginal plus were detected, confirming the ability of adult *Zmynd12*^*–/–*^ males to mate with *Zmynd12*^*+/–*^ and wild-type females. Three males were used in each group, and each male was paired with two females. While the *Zmynd12*^*–/–*^ males were fertile, the average litter size was decreased, suggesting that loss of ZMYND12 can result in male subfertility (Fig. 1H).

### *Zmynd12*^*–/–*^ mice exhibit normal spermatogenesis and spermatozoa morphology

**Fig. 2:**
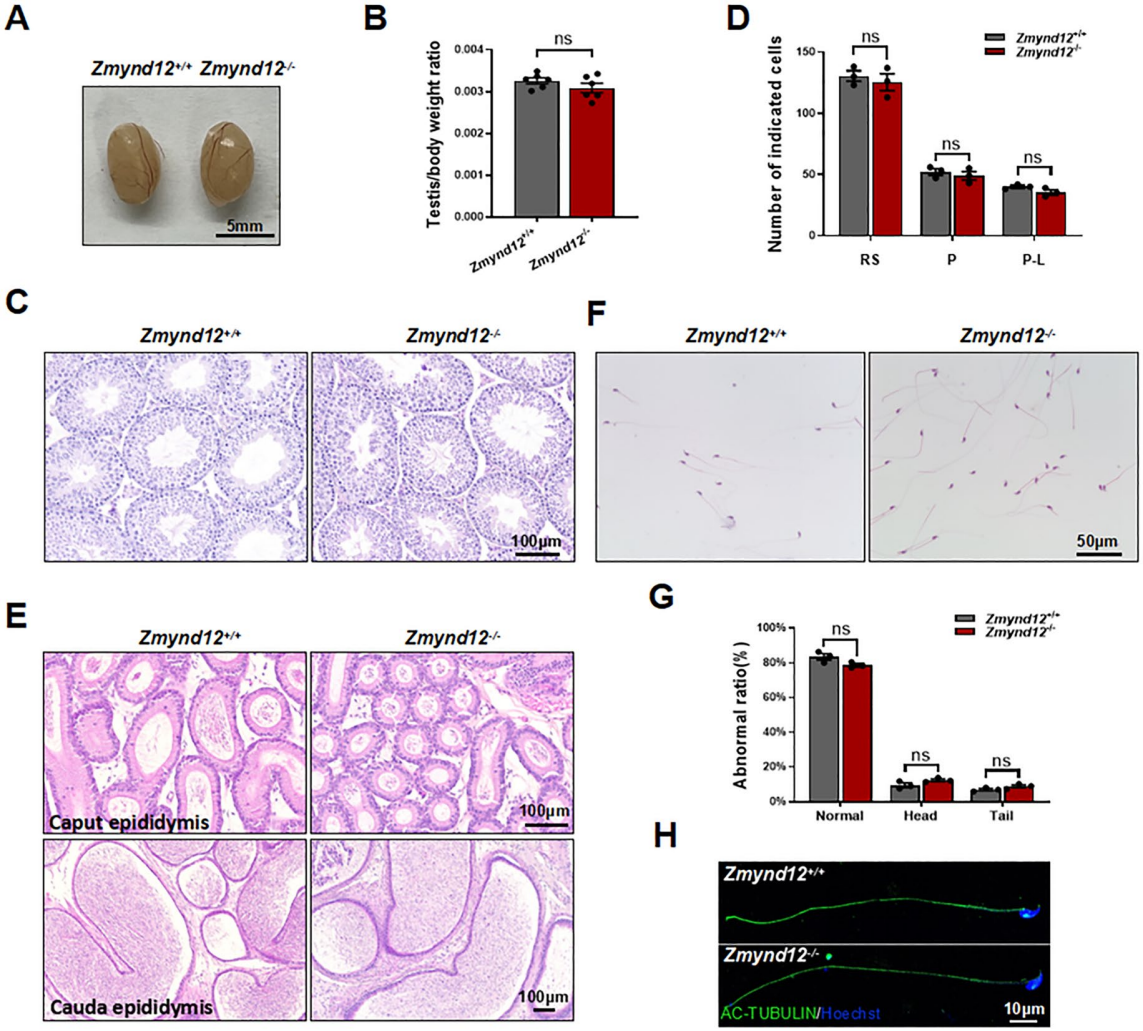
Normal spermatogenic phases are evident in ZMYND12-deficient mice. (**A**) The testes of male *Zmynd12*^+/+^ and *Zmynd12*^−/−^ mice. (**B**) Average testis weights normalized to body weight. Data are means ± SEM, *n* = 6. (**C**) H&E-stained testicular sections from male *Zmynd12*^+/+^ and *Zmynd12*^-/-^ mice, *n* = 3. (**D**) Relative composition ratios for different cell types at spermatogenic stage VIII in the testes of *Zmynd12*^+/+^ and *Zmynd12*^−/−^ mice. P-L, pre-leptotene; P, pachytene; RS, round spermatids. Data are means ± SEM, *n* = 3. (**E**) H&E-stained cauda epididymal sections and caput epididymal sections from male *Zmynd12*^+/+^ and *Zmynd12*^−/−^ mice, *n* = 3. (**F**) H&E-stained spermatozoa from male *Zmynd12*^+/+^ and *Zmynd12*^-/-^ cauda epididymidis, *n* = 3. (**G**) Percentages of spermatozoa from *Zmynd12*^+/+^ and *Zmynd12*^-/-^ mice exhibiting morphological abnormalities. Data are means ± SEM, *n* = 3. (**H**) AC-TUBULIN (green) staining of the spermatozoa from *Zmynd12*^+/+^ and *Zmynd12*^-/-^ mice, *n* = 3

In an effort to explore drivers of subfertility in male *Zmynd12*^*–/–*^ mice, epididymal and testis samples from adult *Zmynd12*^*–/–*^ and control animals were analyzed. No differences in testicular size or appearance were observed when comparing these two groups of mice (Fig. 2A), nor was there any significant difference in the testicular/body weight ratio (Fig. 2B). Hematoxylin and eosin (H&E) staining revealed no evidence of apparent defects in *Zmynd12*^*–/–*^ testis sections (Fig. 2C), and there were also no differences in average spermatocyte, round spermatid, pachytene, or pre-leptotene counts per tubule between these two groups of mice (Fig. 2D; Supplementary Fig. S4A). Furthermore, the flagella of elongated spermatids did not differ between the two genotypes (Supplementary Fig. S4B). Epididymal morphology was similarly normal in these *Zmynd12*^*–/–*^ mice (Fig. 2E). In addition, the *Zmynd12*^*–/–*^ mice were capable of producing sufficient morphologically normal spermatozoa (Fig. 2F-H).

### ZMYND12 is essential for sperm motility

**Fig. 3:**
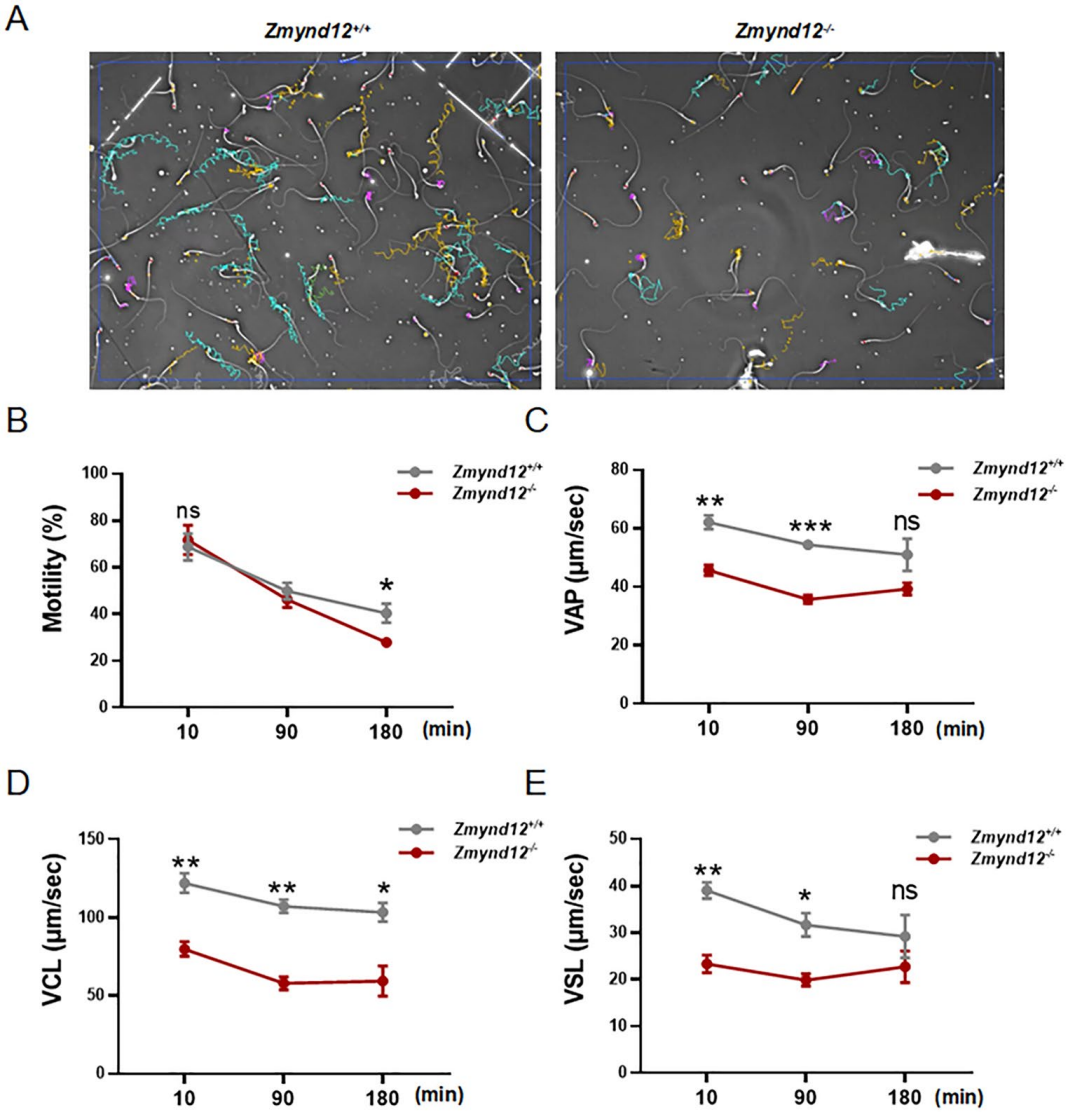
*Zmynd12*^−/−^ mice exhibit impaired swimming parameters. (**A**) Sperm motility tracing performed using a computer-assisted sperm analysis system after incubation for 10, 90, and 180 min, *n* = 3. (**B**) Total cauda epididymal sperm motility for samples from male *Zmynd12*^+/+^ and *Zmynd12*^−/−^ mice were assessed following an incubation period for 10, 90, and 180 min. Data are means ± SEM, *n* = 3. (**C**-**E**) VAP (average path velocity) (**C**), VCL (curvilinear velocity) (**D**), and VSL (straight line velocity) (**E**) were assessed following an incubation period for 10, 90, and 180 min. Data are means ± SEM, *n* = 3

Given that it is an IDA component, ZMYND12 may serve as a regulator of flagellar motility. A computer-assisted sperm analyzer (CASA) was thus used to assess the motility of sperm from prepared knockout mice. While the spermatozoa of *Zmynd12*^*+/+*^ mice were able to move in a linear manner, those of *Zmynd12*^*–/–*^ mice moved with a circular trajectory (Fig. 3A). In addition, the data revealed comparable total motility when comparing the samples from *Zmynd12*^*+/+*^ and *Zmynd12*^*–/–*^ mice (Fig. 3B). However, these *Zmynd12*^*–/–*^ animals did exhibit significant reductions in sperm average path velocity (VAP), curvilinear velocity (VCL), and straight line velocity (VSL) as compared to controls (Fig. 3C-E). When these sperm were cultivated in a 37℃, 5% CO_2_ incubator, the motility of *Zmynd12*^*−/−*^ sperm declined more dramatically relative to WT sperm (Fig. 3B-E). These results suggest a role for ZMYND12, with the loss of this protein potentially contributing to reduced sperm velocity.

### The flagella of ZMYND12-deficient sperm are structurally normal

**Fig. 4:**
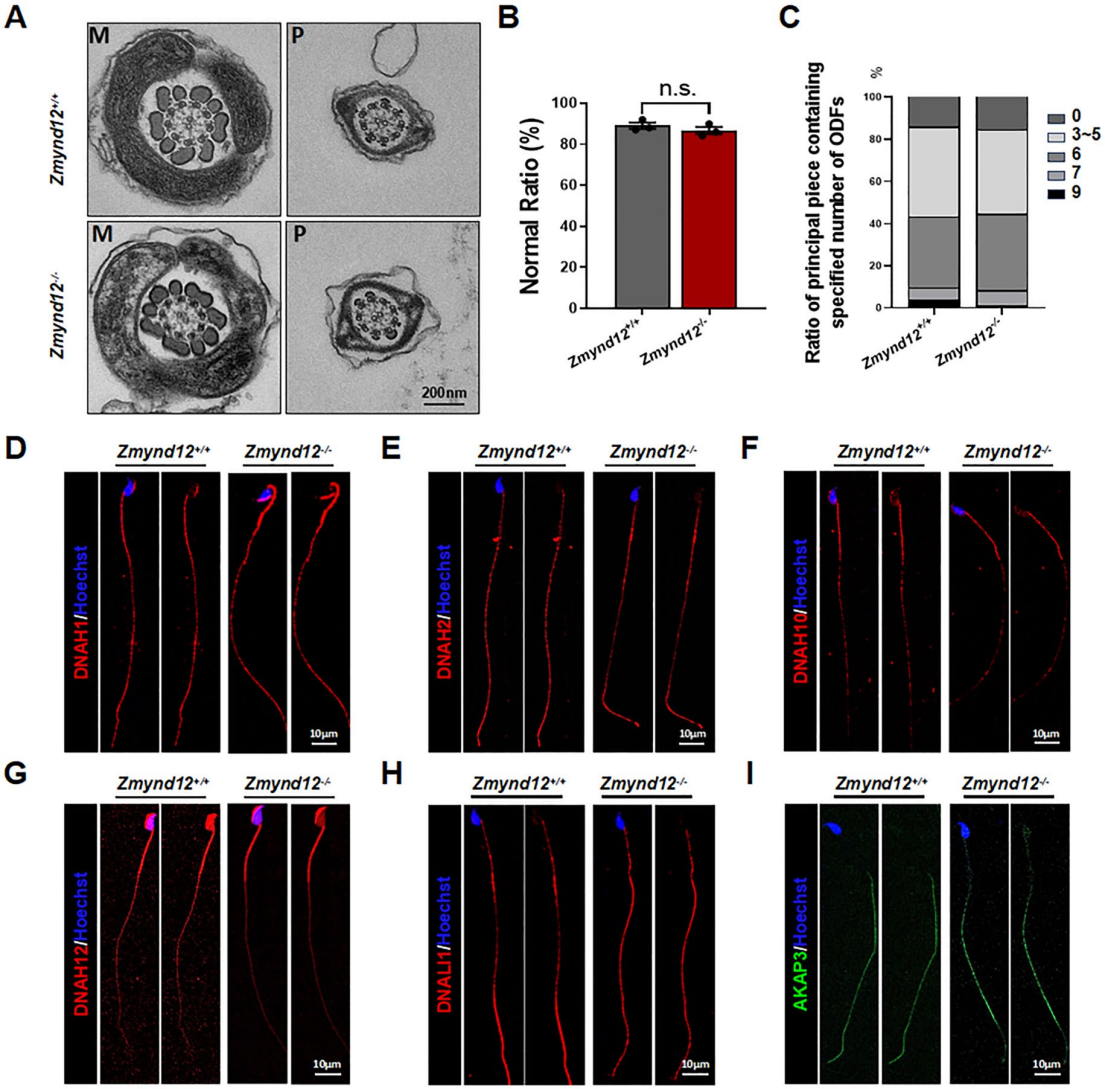
ZMYND12-deficient sperm flagella do not exhibit any substantial abnormalities. (**A**) TEM-based ultrastructural analyses of flagellar cross-sections from the spermatozoa of *Zmynd12*^+/+^ and *Zmynd12*^−/−^ mice. M, mid-piece; P, principal piece. (**B**) Percentages of sperm from *Zmynd12*^+/+^ and *Zmynd12*^−/−^ mice exhibiting ultrastructural abnormalities detected via TEM. Data are means ± SEM, *n* = 3. (**C**) Abnormal ODF quantification for cross-sections of the mid-piece and principal piece of spermatozoa from *Zmynd12*^+/+^ and *Zmynd12*^−/−^mice. Data are means ± SEM, *n* = 3. (**D**-**I**) Immunofluorescent staining was used to detect DNAH1 (**D**), DNAH2 (**E**), DNAH10 (**F**), DNAH12 (**G**), DNALI1 (**H**), and AKAP3 (**I**) in *Zmynd12*^+/+^ and *Zmynd12*^-/-^ spermatozoa, *n* = 4

Flagellar structural integrity is vital for effective sperm motility. As such, transmission electron microscopy was used to evaluate the ultrastructural properties of sperm flagella from *Zmynd12*^*+/+*^ and *Zmynd12*^*–/–*^ mice. The mid-piece of sperm from *Zmynd12*^*–/–*^ animals presented with the expected “9 + 2” axonemal microtubular arrangement, together with IDA, radial spoke, and outer dense fiber structures that were intact and consistent with those of *Zmynd12*^*+/+*^ sperm (Fig. 4A-C). This suggests that *Zmynd12*^*–/–*^ spermatozoa do not present with any pronounced abnormalities, as was subsequently confirmed through immunofluorescent staining.

In *Chlamydomonas* flagella, the homolog of ZMYND12 is a structural component of the IDAd. However, analyses of several known DHCs and other IDA components revealed no abnormalities in any of these cases in samples from *Zmynd12*^*–/–*^ mice, even for the known IDAd component DNAH1 (Fig. 4D-I). These data suggest that the deletion of ZMYND12 does not alter the major structural characteristics of sperm flagella, indicating that ZMYND12 is an accessory IDA subunit the loss of which does not induce significant flagellar abnormalities in murine spermatozoa.

### ZMYND12 influences the localization of PRKACA and regulates sperm capacitation

**Fig. 5:**
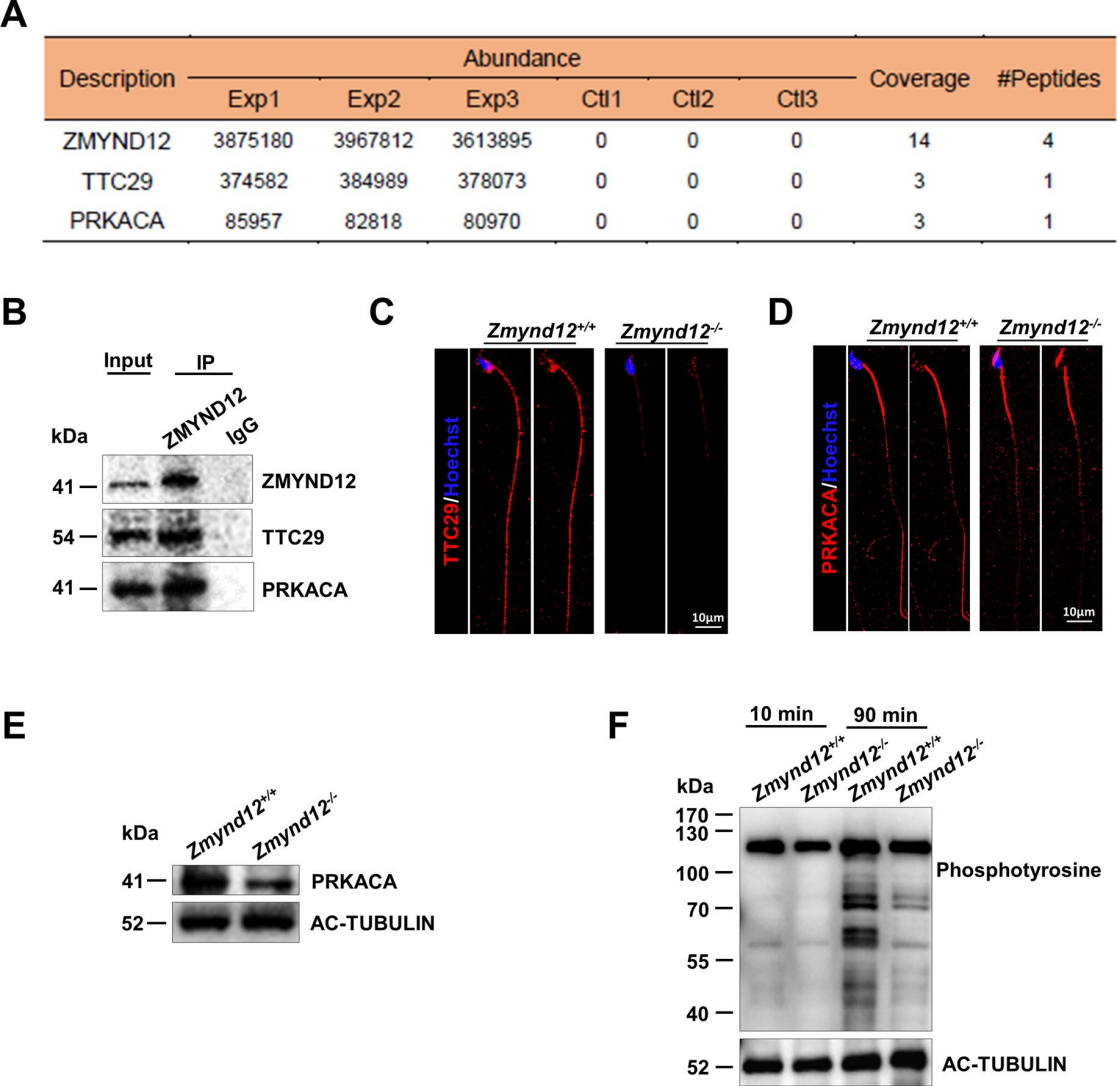
ZMYND12 regulates sperm capacitation and engages in interactions with PRKACA. (**A**) IP was performed using wild-type mouse testis samples, after which silver staining and mass spectrometry were performed, revealing that PRKACA and TTC29 were possible interacting proteins. Exp, experimental group, in which interacting proteins were precipitated with an anti-ZMYND12 antibody; Ctl, control group, where an anti-IgG was used as a negative control for immunoprecipitation; Coverage, the coverage of the identified peptide relative to the protein; #Peptides, the types of peptides identified, *n* = 3. (**B**) Co-immunoprecipitation analysis of the interaction partners of ZMYND12 in testicular protein extracts. (**C**) TTC29 IF staining (red) of *Zmynd12*^+/+^ and *Zmynd12*^−/−^ spermatozoa, *n* = 3. (**D**) PRKACA IF staining (red) of *Zmynd12*^+/+^ and *Zmynd12*^−/−^spermatozoa, *n* = 3. (**E**) Western blotting of PRKACA from *Zmynd12*^+/+^ and *Zmynd12*^−/−^ spermatozoa, with AC-TUBULIN as the internal control, *n* = 3. (**F**) Protein tyrosine phosphorylation associated with capacitation in spermatozoa from both *Zmynd12*^+/+^ and *Zmynd12*^−/−^ mice, with AC-TUBULIN as the internal control, *n* = 3

To further examine the possible mechanisms whereby ZMYND12 can regulate sperm motility, proteins extracted from the testes of adult WT mice were immunoprecipitated in an effort to identify ZMYND12-interacting proteins. Immunoprecipitation was performed using anti-ZMYND12 or anti-IgG as a control (Fig. 5A; Supplementary Table S1). LC-MS/MS analyses of precipitates and western blotting identified PRKACA and TTC29 as candidate ZMYND12 binding partners when assessing only those targets interacting a minimum of three times (Fig. 5A-B). These interactions are partially consistent with results that have been reported in humans [21].

Immunofluorescent staining and western blotting for these candidate ZMYND12 binding partners were next performed on *Zmynd12*^*+/+*^ and *Zmynd12*^*–/–*^ spermatozoa, revealing a significant decrease in PRKACA and TTC29 levels in the absence of functional ZMYND12 (Fig. 5C-E), indicating that the absence of ZMYND12 may affect the assembly of these two proteins. PRKACA is a catalytic subunit of protein kinase A (PKA) known to serve as a regulator of sperm capacitation [24]. Spermatozoa capacitation was next assessed. Western blotting using a specific anti-phosphotyrosine antibody indicated increased protein phosphorylation when wild-type spermatozoa underwent 90 min of in vitro capacitation. However, in contrast, the phosphorylation of *Zmynd12*^*–/–*^ spermatozoa showed significantly reduced compare to the controls *(*Fig. 5F). These results indicate that ZMYND12 may interact with TTC29 and PRKACA. As the ortholog of TTC29 in *Chlamydomonas*, p44, is an IDA component, the interaction between ZMYND12 and TTC29 in mice suggests functional conservation. Additionally, ZMYND12 loss reduced PRKACA levels in the flagellum.

### The brain and tracheal ciliary morphology of *Zmynd12* –/– *mice are normal*

**Fig. 6:**
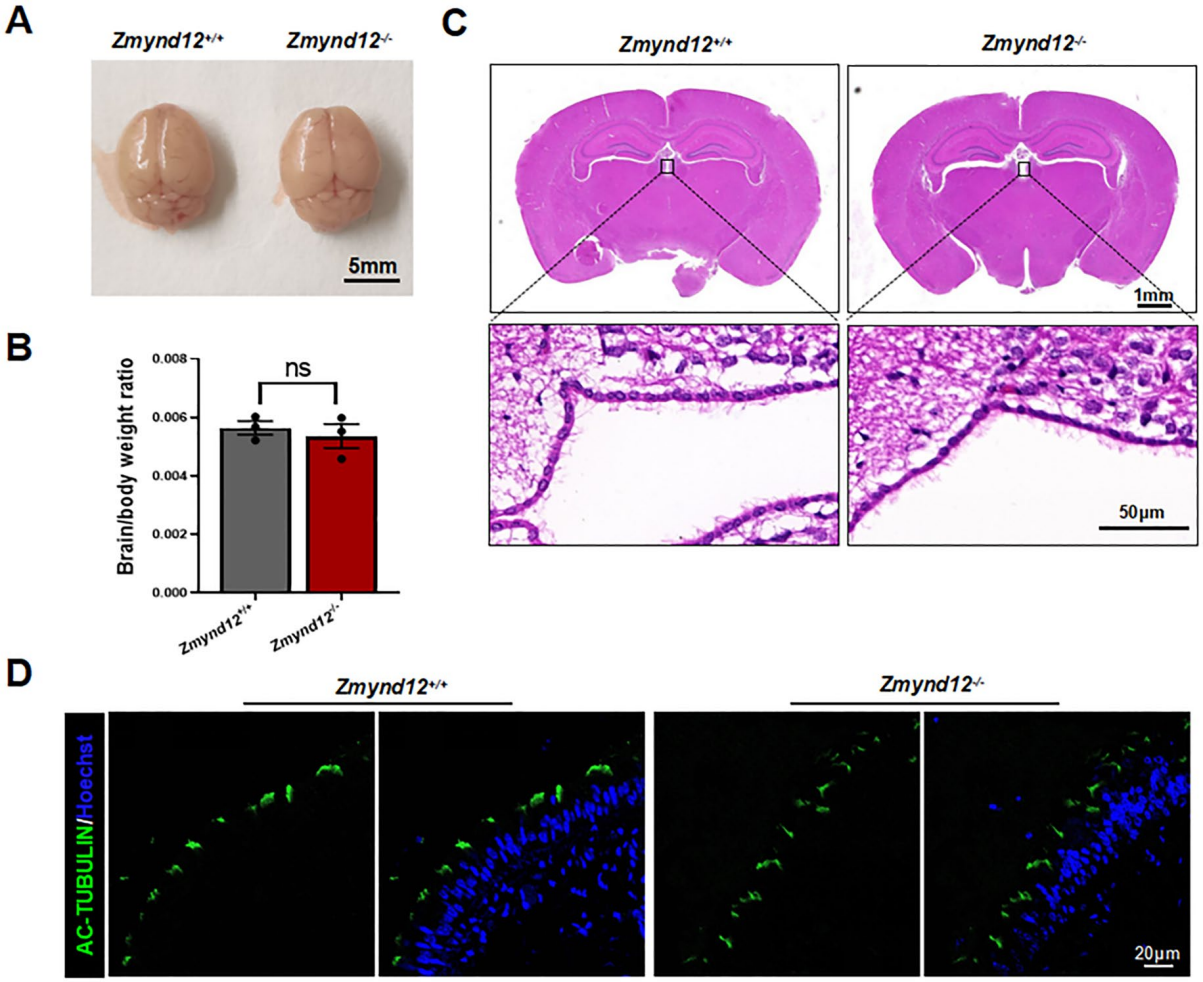
ZMYND12 is not required for normal brain or tracheal cilia morphology. (**A**) Brain images for male *Zmynd12*^+/+^ and *Zmynd12*^−/−^ mice. (**B**) Brain and body weight ratios for male *Zmynd12*^+/+^ and *Zmynd12*^−/−^ mice. Data are means ± SEM, *n* = 3. (**C**) H&E-stained brain sections from male *Zmynd12*^+/+^ and *Zmynd12*^−/−^ mice, *n* = 3. (**D**) AC-TUBULIN IF staining (green) in *Zmynd12*^+/+^ and *Zmynd12*^−/−^ tracheal ciliated columnar epithelial cells, *n* = 3

The “9 + 2” microtubular arrangement is conserved in sperm flagella and in all motile cilia. Furthermore, defects in IDAd have been shown to cause ciliopathies, including primary ciliary dyskinesia (PCD) and MMAF in humans [15, 25, 26]. *Zmynd12*^*–/–*^ mice were next evaluated for any potential PCD-related phenotypes. Initial analyses suggested that the brain samples of these knockout mice exhibited weights and external morphological characteristics consistent with those of *Zmynd12*^*+/+*^ animals (Fig. 6A-B). Consistently, brain sections from these *Zmynd12*^*–/–*^ mice that had been stained with H&E appeared similar to those of WT mice (Fig. 6C).

Further comparisons of the tracheas of *Zmynd12*^*–/–*^ and WT mice revealed no differences in the length of tracheal cilia, nor were there any differences in the expression or localization of AC-TUBULIN in tracheal sections from these animals (Fig. 6D). The loss of ZMYND12 function in mice thus fails to give rise to any apparent morphological defects impacting the brain ventricles and tracheal cilia. Additional research, however, will be necessary to characterize the functional performance of *Zmynd12*^*−/−*^ cilia.

### Identification of biallelic *ZMYND12* variants in a patient with AZS

**Fig. 7:**
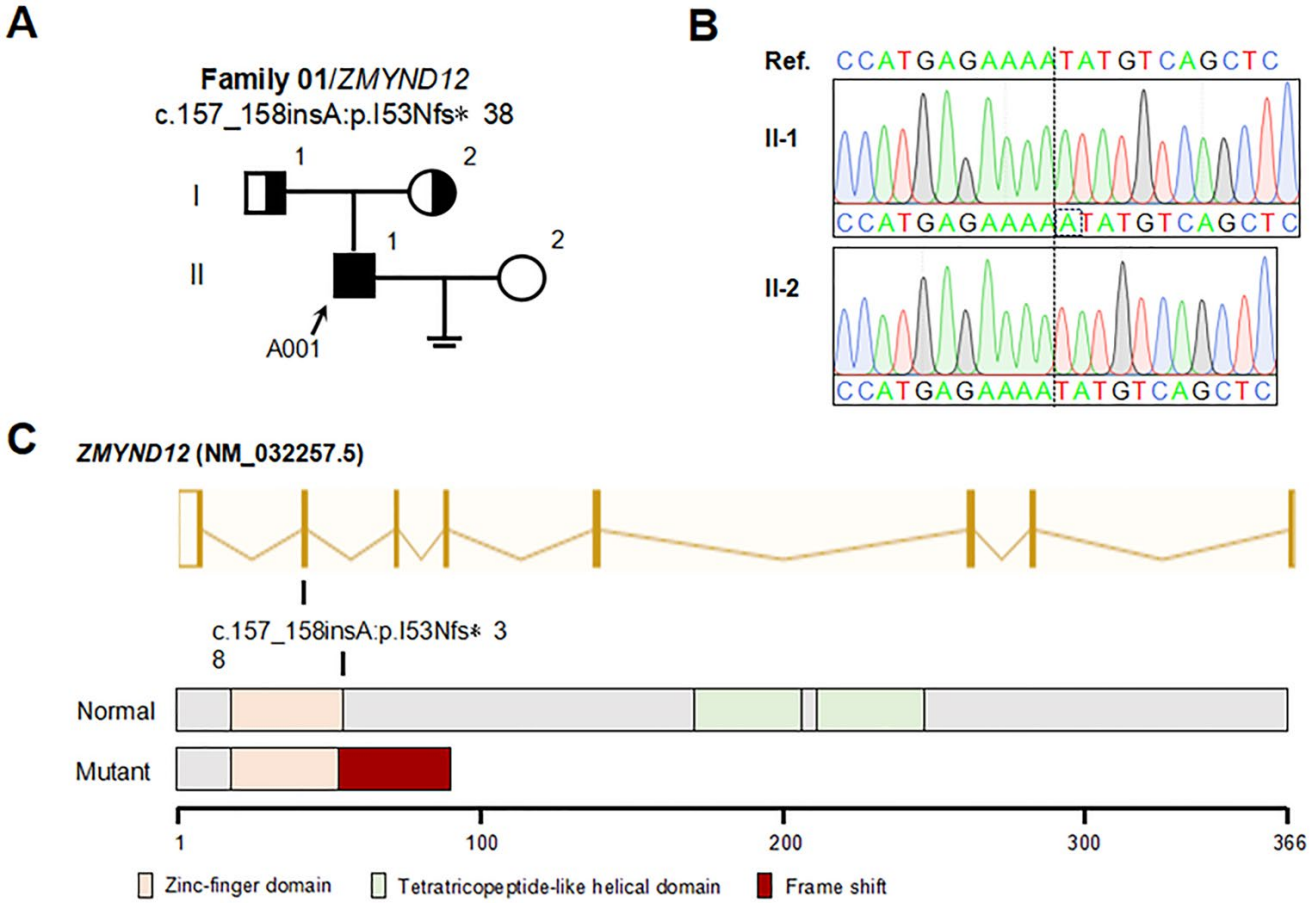
Identification of a ZMYND12 mutation in a male with AZS. (**A**) Pedigree analysis of the family affected by biallelic *ZMYND12* variations. Males suffering from infertility are marked with filled black squares. (**B**) Sanger sequencing was used to verify *ZMYND12* variants identified using whole-exome sequencing in a male with AZS (A001). Inserted bases are marked with a black dashed box (C) The locations of variations in the *ZMYND12* gene

Whole exome sequencing (WES) analyses led to the identification of a biallelic *ZMYND12* variant in a patient with AZS (A001: II-1, 35 years old) (Fig. 7A). Through sanger sequencing, this biallelic *ZMYND12* variant was confirmed to have originated from two asymptomatic heterozygous parents, suggesting that it is subject to autosomal recessive inheritance (Fig. 7B). This variant entailed a 1-bp insertion that was predicted to introduce a translational frameshift and a premature stop codon at position 38 of the 53th ZMYND12 amino acid coding sequence (Fig. 7C). Analyses of semen samples from this proband individual (A001) revealed a total motility of 7.25%, and a progressive motility of 1.5% (Table 1). In line with prior reports [21], this patient presented with a high proportion of morphologically abnormal sperm (Table 1).

**Table 1:** Semen data of the patient

Semen parameters	A001	Normal values
Color	gray-white	Milk-white, gray-white, yellowwish
Semen volume(ml)	3	≥ 1.5
pH	7.4	7.2–8.5
Sperm concentration (M/ml)	7.25	≥ 15
Progressive motility (%)	1.5	≥ 32
Motility	2	≥ 40
Morphologically normal sperm (%)	0	> 4
Short flagella (%)	31.2	< 1.0
Coiled flagella (%)	17.2	< 17.0
Absent flagella (%)	24.7	< 5.0
Angulation (%)	7.5	< 13.0
Irregular calibre (%)	19.4	< 2.0

Normal Values based on the World Health Organization standards and the distribution ranges of morphologically abnormal spermatozoa observed in fertile individuals [27, 28];

M, million

## Discussion

In this study, ZMYND12 was identified as an IDA component in mice that is expressed at the highest levels in male germ cells and that is required for normal sperm motility and capacitation. *Zmynd12*^*–/–*^ male mice exhibited subfertility phenotypes, with reductions in sperm velocity despite the absence of any overt structural defects. The ability of ZMYND12 and TTC29 to interact supports their association with the IDAd subspecies in murine spermatozoa, and further analyses suggested that ZMYND12 may regulate sperm capacitation by influencing PRKACA assembly.

### ZMYND12 serves as a specialized IDAd accessory subunit in mice sperm

Axonemal dynein consists of the ODA and IDA, with the ODA repeating every 24 nm and 7 IDA subspecies repeating in the 96 nm range [29]. IDAs are important for bending motions, whereas ODAs provide acceleration [30]. IDAs have a complex composition, with the major IDA species (a, b, c, d, e, f/I1, and g) exhibiting distinct compositional and location profiles within the axoneme [31]. These IDAs are composed of multiple subunits that are broadly classified into the DHC, LC, IC, and accessory subunit categories [18]. Distal DHCs play a vital role in the conversion of ATP-derived chemical energy into mechanical force, thereby driving ciliary motility [32]. Proximal IC, LC, and LIC light subunits form the foundation of IDA complexes and are thought to regulate dynein activity. Of these, ICs are specific to IDA f/I1, while many other IDA components are believed to perform non-IDA functions. For example, DNALI1 is an IDAd LIC subunit that is also a component of cytoplasmic dynamin and is involved in IMT [1, 33]. Here, ZMYND12 was selected as the component of interest, with its *Chlamydomonas* ortholog, p38, having previously been classified as an IDAd accessory subunit [18]. While the precise functional role of p38 was not established, it was found to localize to the cilia and to play a role in axonemal IDAd docking [34]. Here, TTC29 was identified as an interaction partner of ZMYND12. The finding that the ortholog of TTC29 in *Chlamydomonas* is an IDAd subunit [18] suggests that ZMYND12 may show IDAd localization in mouse sperm.

### ZMYND12 serves as a regulator of PRKACA assembly that induces sperm capacitation

In contrast to their prior characterization as simple, rigid structures, sperm flagella are now understood to be highly complex organelles that contain an array of specialized enzymes. TSSK4, for example, is a Testis-Specific Serine/Threonine Protein Kinase (TSSK) family protein found in the outer dense fibers where it controls the structural organization and motility of sperm through interactions with ODF2 [35]. The fibrous sheath scaffold protein AKAP (A kinase anchoring protein) is capable of binding to protein kinase A (PKA) and particular subcellular substrates to protect the biophosphorylation reaction [36]. The TSSK6 kinase and the DUSP21 phosphatase also exhibit periodic binding activity within the axoneme of murine spermatozoa [37]. Here, an interaction between ZMYND12 and PRKACA was detected such that ZMYND12 deletion resulted in a pronounced drop in PRKACA levels. This supports a role for ZMYND12 as a regulator of the assembly of PRKACA, potentially by serving as an anchoring site for the binding of this kinase, which is important for sperm capacitation. Consistently, a significant reduction in *Zmynd12*^*−/−*^ sperm capacitation was observed in this study, highlighting a novel function for IDAd.

### ZMYND12 exhibits species-specific differences in functionality between mice and humans

To date, many different dynein-associated genes have been established as candidate factors related to male infertility characterized by impaired sperm motility. The loss of DNAH1 function was the first such variant that was conclusively identified as a cause of MMAF cases of AZS [15]. More recently, studies have documented links between male fertility and a range of dynein proteins including DNAH2, with many variants in these genes having been linked to MMAF symptoms [38]. A recent report documented an association between MMAF incidence and *ZMYND12* truncating and frameshift variants [21]. In the present study, a novel variant in *ZMYND12* associated with loss of function was identified in an MMAF patient with high sperm malformation rates. These results highlight the potential pathogenic effects of the loss of ZMYND12 as a driver of male infertility, extending the known spectrum of AZS causes and therefore providing potential benefits to the genetic counseling and healthcare management of individuals found to harbor this genetic variant.

With progressive advances in the understanding of the genetic basis for male infertility, a growing number of animal models have been developed to confirm and characterize the pathogenicity of certain variants in rodents and primates [39, 40]. The knockdown of the *ZMYND12* ortholog TbTAX-1 in *Trypanosoma brucei*, has been reported to markedly alter flagellar motility in a manner akin to the changes evident in the sperm of males bearing homozygous *ZMYND12* variants [21]. While the male knockout mice in this study showed signficantly reduced sperm movement velocity, no corresponding reduction in the total fresh sperm motility was observed. Moreover the sperm from these *Zmynd12*^*−/−*^ mice did not exhibit any overt morphological abnormalities in contrast to the findings from the evaluated human patient. This may suggest that ZMYND12 plays distinct roles in the flagella of sperm from humans and mice. Interestingly, TTC29, as an interaction partner of ZMYND12, has also been found to function differently in humans and mice. Multiple case studies have demonstrated that loss of TTC29 leads to MMAF in humans, while TTC29-deficient mouse sperm showed only subtle morphological defects [41, 42]. As in *Chlamydomonas*, TTC29 and ZMYND12 may function as accessory units in IDAd in mouse sperm, while in human sperm, they play a structural role in the flagella [18]. These differences reflect the functional differences in IDAd between human and mouse sperm flagella. In conclusion, the present data offer clear evidence that ZMYND12 is required for sperm motility and capacitation in mice. The multiple documented cases of human *ZMYND12* variants also support a potential link between variations in this gene and the incidence of MMAF, which is a specific AZS subtype. At the molecular level, ZMYND12 was identified as a binding partner for PRKACA, and TTC29, serving as a key regulator of the functionality of murine flagella.

## Methods

### Animal care and ethics

Mice were housed under specific pathogen-free conditions in a controlled setting (50–70% humidity, 20–22 °C, 12 h light/dark cycle) with free food and water access. Suffering was minimized wherever possible, and mice were sacrificed by cervical dislocation when necessary. Male mice that were 8–10 weeks old were used when harvesting sperm, testes, and brain tissue samples to conduct phenotypic analyses. Sample sizes were not predetermined using any statistical techniques. Mice were assigned to experimental groups at random, and animal studies did not employ any blinding method or exclusion criteria. At the end of the study, all remaining animals were euthanized and used for tissue analyses.

### Study patients

The AZS patients tested in this study were recruited from the Ningbo Women and Children’s Hospital. Participants with abnormalities in somatic chromosome karyotypes, genomic azoospermia factor deletions, serum sex hormone levels, and scrotal ultrasonography were excluded from the analysis. This investigation received ethical approval (approval no. EC2020-048) from the above institution and all subject provided written informed consent prior to the initiation of the study. All protocols were conducted in accordance with the Declaration of Helsinki and approved by the institutional ethics review board.

### Genetic analysis

Whole-exome sequencing and bioinformatic analyses were performed as previously described [1]. Briefly, the extraction of genomic DNA and whole-exome enrichment were performed sequentially, according to a standardized protocol. Subsequently, high-throughput sequencing of the captured DNA was performed on the HiSeq X-TEN or NovaSeq 6000 platforms (Illumina, San Diego, CA, USA). Standard assembly (Burrows–Wheeler Aligner, http://bio-bwa.sourceforge.net/), calling (Genome Analysis Toolkit, https://gatk.broadinstitute.org/hc/en-us), and annotation (ANNOVAR, https://annovar.openbioinformatics.org/en/latest/) were then performed. Lastly, Sanger sequencing was conducted to verify the candidate mutations and corresponding origins.

### CRISPR/Cas9-mediated knockout

*Zmynd12*^−/−^ mice were produced using a CRISPR/Cas9-based approach. Briefly, guide RNAs (gRNAs) targeting exon 2–7 of *Zmynd12* were created (gRNA1: 5′-GTAAGTCCACATACCCACAAAGG-3′ and gRNA2: 5′-GCTGCCACGTCAGCCTACACAGG-3′). Zyotes from C57BL/6 mice were simultaneously injected with these gRNAs and Cas9 mRNA, after which the embryos were transferred into the uterus of pseudopregnant recipient female mice. The genotypes of offspring were then confirmed through PCR amplification with primers detailed in Supplementary Table S2.

### qPCR

Trizol (Thermo Fisher Scientific, 15,596,026) was used to extract total RNA from each sample, of which 1 µg per sample was then reverse transcribed with the PrimeScript™ RT reagent Kit (Takara, RR036A) based on provided directions to generate cDNA. SYBR Green Master Mix (Vazyme, Q131) and a LightCycler480II system (Roche) were then used for all qPCR analyses performed with primers shown in supplementary Table S2, with 18 S rRNA serving as a reference control.

### Western immunoblotting

After extracting proteins with RIPA buffer (Beyotime, P0013B) and quantifying their levels with a BCA Kit (Beyotime, P0012), equal protein amounts were separated via 10% SDS-PAGE and transferred to PVDF membranes. Blots were then blocked with 5% BSA (Sigma, v900933) in TBS for 2 h at room temperature, followed by overnight incubation with appropriately diluted primary antibodies (4 °C, overnight). Blots were blocked four times using TBST (15 min/wash), followed by incubation with horseradish peroxidase (HRP)-conjugated secondary antibodies (2 h, room temperature. A chemiluminescence reagent was then used for protein band detection.

### Antibodies

Purchased antibodies included anti-ZMYND12 used for IP, WB and IF (Proteintech, 25587-1-AP), anti-PRKACA used for WB and IF (Proteintech, 24503-1-AP), anti-TTC29 used for WB and IF (Atlas Antibodies, HPA061473), anti-Phosphotyrosine used for WB (Merck Millipore, 05-1050X), anti-AC-TUBULIN used for WB and IF (FineTest, FNab00082), anti-DNAH1 used for IF (Thermo Fisher Scientific, PA5-57826), anti-DNAH2 used for IF (Novus, NBP2-49506), anti-DNAH10 used for IF (Bioss, bs-11022R), anti-DNAH12 used for IF (Thermo Fisher Scientific, PA5-63952), anti-DNALI1 used for IF (Proteintech, 17601-1-AP), Anti-γH_2_AX used for IF (Abcam, ab81299), Normal Rabbit IgG used for IP (Cell Signaling Technology, 2729 S), Goat-anti-Mouse IgG (H + L)-HRP used for WB (Beyotime, A0216), Goat-anti-Rabbit IgG (H + L)-HRP used for WB (Beyotime, A0208), IPKine HRP, Mouse Anti-Rabbit IgG LCS used for WB (Abbkine, A25022), Donkey-anti-Mouse IgG, Alexa Fluor488 used for IF (Thermo Fisher Scientific, A-21,202), Donkey-anti-Rabbit IgG, Alexa Fluor555 used for IF (Thermo Fisher Scientific, A-31,572), Donkey-anti-Rabbit IgG, Alexa Fluor488 used for IF (Thermo Fisher Scientific, A-21,206), and the anti-AKAP3 used for IF was a gift from Qi’s lab [43]. The working concentrations of the antibodies are shown in Supplementary Table S3.

### Fertility testing

Fertility analyses were performed by mating sexually mature knockout male mice with two wild-type C57BL/6 female mice for a 6-month period during which the female mice were exchanged every other gestation cycle. Knockout male mice and controls were fed under identical conditions, and litter sizes were recorded during fertility testing. All fertility testing was conducted using 8 to 10-week-old mice.

### Silver staining and LC-MS/MS

After separating proteins by 12% SDS-PAGE, they were stained with a Fast Silver Stain Kit (Beyotime, P0017S). Bands of interest were then excised manually, digested using sequencing-grade trypsin (Promega, WI, USA), and the peptides therein were extracted, dried, and analyzed via LC-MS/MS.

The IP precipitates were separated on SDS-PAGE and stained with AgNO_3_. The bands were removed from the gels following trypsin digestion. The EASY-nanoLC 1200 system (Thermo Fisher Scientific), equipped with an Orbitrap Q Exactive HFX mass spectrometer (Thermo Fisher Scientific) and a nanospray ion source, was used for LC-MS/MS analysis. Mixtures of tryptic peptides were dissolved in 0.1% formic acid (FA) in LC-grade water and injected into an analytical column (75 μm× 25 cm, C18 column, 1.9 μm, Dr. Maisch). Solution A was 0.1% FA and solution B was 80% ACN and 0.1% FA. A 95-min linear gradient (3–5% B for 5 s, 5–15% B for 40 min, 15–28% B for 34 min and 50 s, 28–38% B for 12 min, 30–100% B for 5 s, and 100% B for 8 min) was applied using a high-resolution MS pre-scan, with a mass range of 350–1500. The normalized collision energy for elevated energy collision-driven dissociation (HCD) was adjusted to 28, and the resulting fragments were identified using a resolution of 15,000. All ions chosen for fragmentation were excluded for 30 s via dynamic exclusion. Data processing was done with Proteome Discoverer software (Thermo Fisher Scientific), and the mouse reference proteome was retrieved from the UniProt database (release 2021.04) using standard variables.

### Co-immunoprecipitation

RIPA buffer (1 mL; Beyotime, P0013C) was used to extract total testicular proteins, followed by centrifugation (40 min, 13,000 rpm). Supernatants were then collected, precleared for 1 h using 30 µL of protein A/G beads (Bimake, B23202) at 4 °C, and the lysates were then incubated overnight at 4 °C with appropriate antibodies. Protein complexes were then combined with 60 µL of Protein A/G magnetic beads, followed by a further 6 h incubation at 4 °C. Supernatants were then removed, and beads were washed with RIPA buffer 5 times, followed by the addition of SDS loading buffer. Samples were then boiled for 10 min at 95 °C and dentured proteins were separated by SDS-PAGE and detected with appropriate antibodies. As a negative control, rabbit IgG was also used for co-immunoprecipitation.

### Histological and immunofluorescent staining

Sperm samples were fixed with 4% paraformaldehyde (PFA) for 10 min before spreading on slides. The slides were dried and then rinsed three times with PBS. For the preparation of paraffin-embedded sections, tissues were fixed using 4% paraformaldehyde or modified Davidson’s fluid (MDF) for 48 h. Then, these samples were treated with a gradient of 70%, 80%, 90%, and 100% ethanol, a 1:1 mixture of ethanol and xylene, and pure xylene. After embedding these samples in paraffin, 5 μm sections were cut. Before staining, sections were deparaffinized and rehydrated. For H&E staining, these tissues were strained with hematoxylin and eosin staining solution. For IF staining, sections were treated with 10 mM citrate solution (pH 6) while heating for antigen retrieval. Both the sperm samples and sections were blocked using 1% BSA (Sigma, v900933), followed by overnight incubation at 4 °C with appropriate primary antibodies, washed, and treated for 2 h with secondary antibodies and Hoechst 33,342 at room temperature. Samples were then fixed using glycero, covered using glass coverslips, followed by imaging with an LSM980 confocal microscope (Carl Zeiss).

#### Transmission electron microscopy (TEM)

For TEM, samples were fixed with 1% osmium tetroxide and dehydrated with an ethanol gradient (50, 70, 90, and 100% ethanol) and 100% acetone. After infiltration with acetone and SPI-Chem resin and embedding with Epon 812, the samples were sectioned using an ultra-microtome and stained with uranyl acetate and lead citrate. A JEM-1400 transmission electron microscope (JEOL) was used for sample evaluation and imaging.

### Sperm motility analyses

When analyzing sperm motility, an approach reported previously was employed [44]. Briefly, following the resection of the cauda epididymis from an adult mouse, sperm were dislodged by squeezing into modified HTF medium (Irvine Scientific, 90,126) containing 10% fetal bovine serum (FBS) and incubated at 37℃ for 10 min. The suspended sperm were then assessed with a computer-assisted sperm analysis (CASA, CEROS v.12, Hamilton Thorne Research), allowing for analyses of motile sperm.

#### Capacitation and tyrosine phosphorylation detection in mice sperm

Sperm capacitation and tyrosine phosphorylation detection were conducted based on a published protocol with minor modifications [45, 46]. The excised cauda epididymis was placed in HTF medium, consisting of 101.6 mM NaCl, 4.7 mM KCl, 0.37 mM K_2_PO_4_, 0.2 mM MgSO_4_·7H_2_O, 2 mM CaCl_2_, 25 mM NaHCO_3_, 2.78 mM glucose, 0.33 mM pyruvate, 21.4 mM sodium lactate, 286 mg/L penicillin G, 228 mg/L streptomycin, and 5 mg/ml fatty acid-free BSA (Sangon, A602448), to release sperm. The sperm were divided into non-capacitated and capacitated groups, with the latter incubated at 37 °C with 5% CO2 for 90 min. After centrifugation at 500 g and 4 °C, the pellet was collected. The proteins were extracted and subjected to Western blot analysis of tyrosine phosphorylation.

### Statistical analysis

Student’s two-tailed t-tests were used to compare data in GraphPad Prism. Not significant (ns), *P* ≥ 0.05; *P < 0.05, **P < 0.01, ***P < 0.001, ****P < 0.0001. The exact sample sizes (n) for each experimental group/condition are provided in the figure legends.All analyses were performed at least in triplicate.

### Electronic supplementary material

Below is the link to the electronic supplementary material.

Supplementary Material 1

Supplementary Material 2

Supplementary Material 3

Supplementary Material 4

## Data Availability

All data relevant to the study are included in the article or uploaded as supplementary information.
