# Supplementary material for: ZMYND12 serves as an IDAd subunit that is essential for sperm motility in mice
Source: Cell Mol Life Sci. 2024 Jul 27;81(1):317. doi: 10.1007/s00018-024-05344-7 (PMC11335240; doi:10.1007/s00018-024-05344-7)
Supplement: Supplementary file 1 — Supplementary Material 1 [file 18_2024_5344_MOESM1_ESM.docx]

**Supplementary Information**

**Supplementary figures**


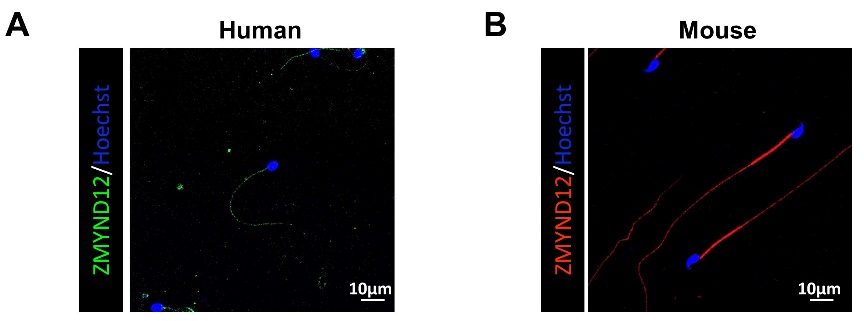


**Figure S1.** Immunofluorescence (IF) staining for ZMYND12 in spermatozoa from normal humans (A) and wildtype mice (B) performed with anti-ZMYND12, n=3.

**
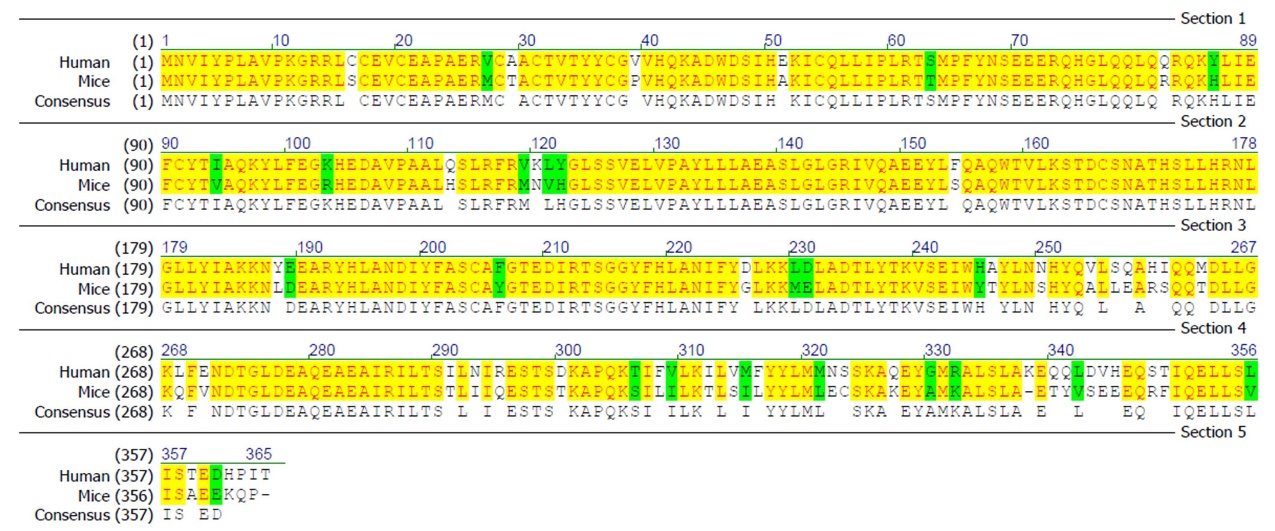
**

**Figure S2.** ZMYND12 is a highly conserved gene in human and mice.


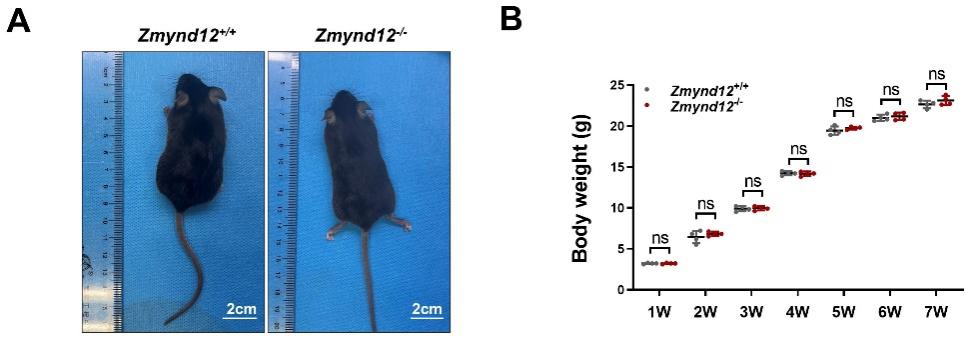


**Figure S3. ZMYND12-deletion does not affect mouse growth.** (A) Representative images of *Zmynd12*^+/+^ and *Zmynd12*^-/-^ males at 8 weeks old. (B) Body weights of *Zmynd12*^+/+^ and *Zmynd12*^-/-^ males at the indicated ages, n=4.


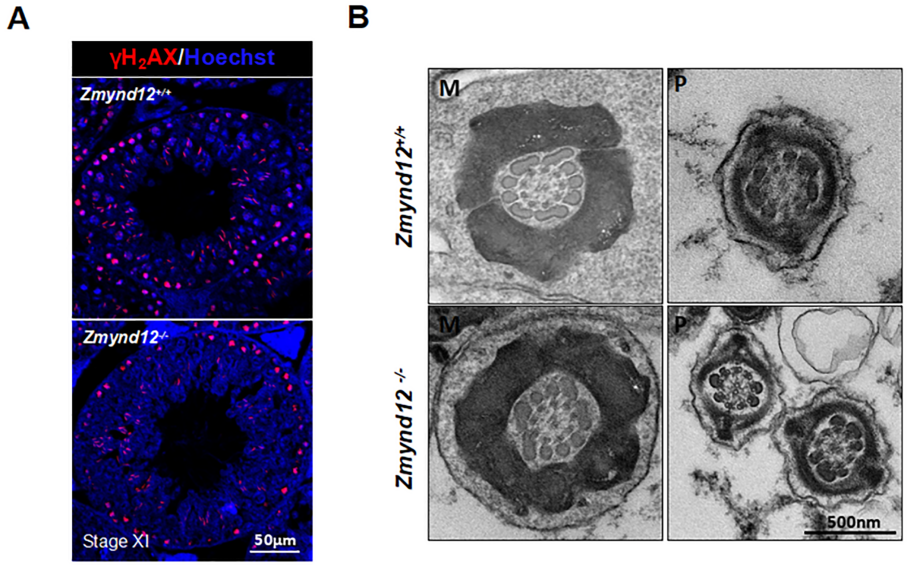


**Figure S4. *Zmynd12*^-/-^ mice exhibit normal fertility phenotypes** (A) IF staining of γH_2_AX in *Zmynd12*^+/+^ and *Zmynd12*^-/-^ testes, n=3. The γH_2_AX signal in the spermatocytes and elongating spermatids were shown. (B) The ultrastructure of testicular elongating spermatids. M, mid-piece; P, principal piece.
