# Supplementary material for: ZMYND12 serves as an IDAd subunit that is essential for sperm motility in mice
Source: Cell Mol Life Sci. 2024 Jul 27;81(1):317. doi: 10.1007/s00018-024-05344-7 (PMC11335240; doi:10.1007/s00018-024-05344-7)
Supplement: Supplementary file 3 — Supplementary Material 3 [file 18_2024_5344_MOESM3_ESM.doc]

| **Primer name** | **Primer sequence(5'-3')** | **Expected product size (bp)** |
| --- | --- | --- |
| Mus-Zmynd12-F1 | CATTCCCTTCCTTTCTTCCTGCTG | 570 |
| Mus-Zmynd12-R1 | ATAACCAAACCTTGTGTGCTTCAG |  |
| Mus-Zmynd12-R2 | ATAACAAATCACACCGGGTTAGCA | 843 |
| Mus-Zmynd12-F | CCTCAGGAGGCTACTTCCAC | 126 |
| Mus-Zmynd12-R | GGGCTTGATAGTGACTGTTGAGA |  |
| Mus-18S-F | TAACGAACGAGACTCTGGCAT | 138 |
| Mus-18S-R | CGGACATCTAAGGGCATCACAG |  |

**Supplementary Table S1. Primers used in PCR and RT-qPCR**
